# V_1.42_In_1.83_Mo_15_Se_19_

**DOI:** 10.1107/S1600536810036834

**Published:** 2010-09-18

**Authors:** Patrick Gougeon, Philippe Gall, Diala Salloum, Michel Potel

**Affiliations:** a Sciences Chimiques de Rennes, UMR CNRS No. 6226, Université de Rennes I – INSA Rennes, Avenue du Général Leclerc, 35042 Rennes CEDEX, France

## Abstract

The structure of the title compound, vanadium indium penta­deca­molybdenum nona­deca­selenide, V_1.42_In_1.83_Mo_15_Se_19_, is isotypic with In_2.9_Mo_15_Se_19_ [Grüttner *et al.* (1979 ▶). *Acta Cryst.* B**35**, 285–292]. It is characterized by two cluster units Mo_6_Se^*i*^_8_Se^*a*^_6_ and Mo_9_Se^*i*^_11_Se^*a*^_6_ (where *i* represents inner and *a* apical atoms) that are present in a 1:1 ratio. The cluster units are centered at Wyckoff positions 2*b* and 2*c* and have point-group symmetry 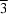 and 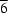, respectively. The clusters are inter­connected through additional Mo—Se bonds. In the title compound, the V^3+^ cations replace the trivalent indium atoms present in In_2.9_Mo_15_Se_19_, and a deficiency is observed on the monovalent indium site. One Mo, one Se and the V atom are situated on mirror planes, and two other Se atoms and the In atom are situated on threefold rotation axes.

## Related literature

For previous reports on the crystal structure of In_∼3_Mo_15_Se_19_ compounds, see: Grüttner *et al.* (1979 ▶). For physical properties of this type of compound, see: Seeber *et al.* (1979 ▶). The crystal structures of the substituted compounds Ho_0.76_In_1.68_Mo_15_Se_19_ and In_0.87_K_2_Mo_15_Se_19_ were reported by Salloum *et al.* (2006 ▶; 2007 ▶). For details of the *i*- and *a*-type ligand notation, see: Schäfer & von Schnering (1964 ▶).

## Experimental

### 

#### Crystal data

V_1.42_In_1.83_Mo_15_Se_19_
*M* *_r_* = 3221.80
Hexagonal, 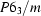

*a* = 9.7361 (1) Å
*c* = 19.3090 (4) Å
*V* = 1585.11 (4) Å^3^
*Z* = 2
Mo *K*α radiation
μ = 29.21 mm^−1^
*T* = 293 K
0.09 × 0.07 × 0.05 mm

#### Data collection

Nonius KappaCCD diffractometer
Absorption correction: analytical (de Meulenaar & Tompa, 1965 ▶) *T*_min_ = 0.161, *T*_max_ = 0.329
27560 measured reflections
2390 independent reflections
1634 reflections with *I* > 2σ(*I*)
*R*_int_ = 0.093

#### Refinement

*R*[*F* ^2^ > 2σ(*F* ^2^)] = 0.037
*wR*(*F* ^2^) = 0.087
*S* = 1.08
2390 reflections
67 parameters
Δρ_max_ = 3.22 e Å^−3^
Δρ_min_ = −2.57 e Å^−3^

### 

Data collection: *COLLECT* (Nonius, 1998 ▶); cell refinement: *COLLECT*; data reduction: *EVALCCD* (Duisenberg *et al.*, 2003 ▶); program(s) used to solve structure: *SIR97* (Altomare *et al.*, 1999 ▶); program(s) used to refine structure: *SHELXL97* (Sheldrick, 2008 ▶); molecular graphics: *DIAMOND* (Bergerhoff, 1996 ▶); software used to prepare material for publication: *SHELXL97*.

## Supplementary Material

Crystal structure: contains datablocks I, global. DOI: 10.1107/S1600536810036834/wm2403sup1.cif

Structure factors: contains datablocks I. DOI: 10.1107/S1600536810036834/wm2403Isup2.hkl

Additional supplementary materials:  crystallographic information; 3D view; checkCIF report

## Figures and Tables

**Table 1:** Selected bond lengths (Å)

Mo2—Se5	2.5259 (8)
Mo2—Se2	2.5974 (6)
Mo2—Se2^i^	2.6290 (6)
Mo2—Mo2^i^	2.6474 (7)
Mo2—Se1^ii^	2.6504 (6)
Mo2—Se3^i^	2.6966 (6)
Mo2—Mo3^i^	2.7136 (5)
Mo2—Mo3	2.7557 (5)
Mo1—Se1^iii^	2.5467 (7)
Mo1—Se4	2.5481 (8)
Mo1—Se1	2.5717 (6)
Mo1—Se1^iv^	2.6111 (6)
Mo1—Se2	2.6378 (7)
Mo1—Mo1^v^	2.6992 (7)
Mo1—Mo1^vi^	2.7223 (8)
Mo3—Se2	2.5631 (6)
Mo3—Se2^vii^	2.5631 (6)
Mo3—Se3	2.5965 (9)
Mo3—Se3^i^	2.6095 (9)
Mo3—Mo3^viii^	2.7056 (10)
Mo3—Mo2^ix^	2.7136 (5)
In1—Se5	3.0759 (15)
In1—Se2^x^	3.1221 (5)
In1—Se1^x^	3.4904 (7)
V1—Se4	2.510 (2)
V1—Se3^iv^	2.730 (3)
V1—Se2^v^	2.7981 (18)
V1—Se3^v^	2.831 (3)
V1—Mo3^v^	2.865 (3)

Symmetry codes: (i) 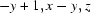; (ii) 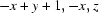; (iii) 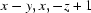; (iv) 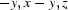; (v) 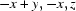; (vi) 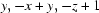; (vii) 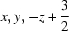; (viii) 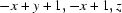; (ix) 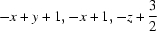; (x) 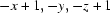.

**Fig. 1.**
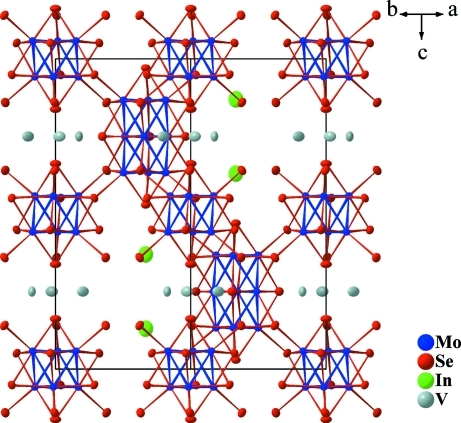
View of V1.42In1.83Mo15Se19 along [110]. Displacement ellipsoids are drawn at the 97% probability level.

**Fig. 2.**
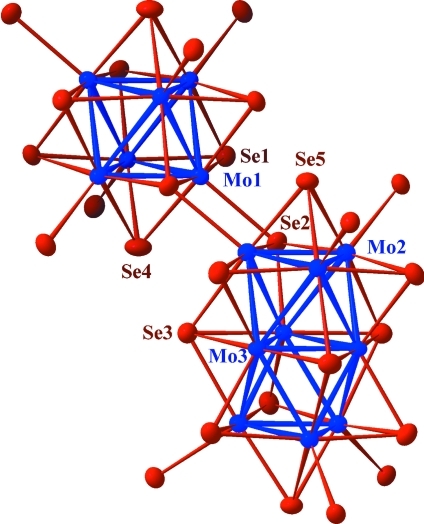
Plot showing the atom-numbering scheme and the interunit linkage of the Mo9Se11Se6 and Mo6Se8Se6 cluster units. Displacement ellipsoids are drawn at the 97% probability level.

## supplementary crystallographic information

### Comment

From the point of view of crystal chemistry and physical properties, the reduced molybdenum selenides In_~3_Mo_15_Se_19_ (Grüttner *et al.*, 1979) constitute an interesting family of compounds. Indeed, their crystal structure contains an equal mixture of Mo_6_ and Mo_9_ cluster units and the In atoms occupy two crystallographically different positions depending on their formal oxidation state of +1 or +3. Recently, we have shown that the In^3+^ cation can be replaced by other trivalent cations such as Ho^3+^ (Salloum *et al.*, 2006) and the In^+^ cation by K^+^ (Salloum *et al.*, 2007). Interest in these Mo cluster compounds also lies in their physical properties, because they become superconductors with high critical magnetic fields at about 4 K (Seeber *et al.*, 1979). We present here the crystal structure of V_1.42_In_1.83_Mo_15_Se_19_ in which a 3*d* element replaces the trivalent indium atom.

The Mo—Se framework of the title compound consists of the cluster units Mo_6_Se*^i^*_8_Se*^a^*_6_ and Mo_9_Se*^i^*_11_Se*^a^*_6_ in a 1:1 ratio (for details of the *i*- and *a*-type ligand notation, see: Schäfer & von Schnering (1964)). Both cluster units are interconnected through additional Mo—Se bonds (Figs. 1 and 2). The first unit can be described as an Mo_6_ octahedron surrounded by eight face-capping inner Se*^i^* and six apical Se*^a^* ligands. The Mo_9_ cluster is surrounded by 11 Se*^i^* atoms capping one or two faces of the bioctahedron and six Se*^a^* ligands above the apical Mo atoms. The Mo_6_Se*^i^*_8_Se*^a^*_6_ and Mo_9_Se*^i^*_11_Se*^a^*_6_ units are centered at Wyckoff positions 2*b* and 2*c* and have point-group symmetry 3 and 6, respectively. The Mo—Mo distances within the Mo_6_ cluster are 2.6992 (7) Å for the distances of the Mo triangles formed by the Mo1 atoms related through the threefold axis, and 2.7223 (8) Å for the distances between these triangles. The Mo—Mo distances within the Mo_9_ clusters are 2.6474 (7) and 2.7056 (10) Å in the triangles formed by the atoms Mo2 and Mo3, respectively, and 2.7136 (5) and 2.7557 (5) Å for those between the Mo2_3_ and Mo3_3_ triangles. The Se atoms bridge either one (Se1, Se2, Se4 and Se5) or two (Se3) triangular faces of the Mo clusters. Moreover, atoms Se1 and Se2 are linked to an Mo atom of a neighboring cluster. The Mo—Se bond lengths range from 2.5467 (7) to 2.6378 (7) Å within the Mo_6_Se*^i^*_8_Se*^a^*_6_ unit, and from 2.5259 (8) to 2.6966 (6) Å within the Mo_9_Se*^i^*_11_Se*^a^*_6_ unit. Each Mo_9_Se*^i^*_11_Se*^a^*_6_ cluster is interconnected by six Mo_6_Se*^i^*_8_Se*^a^*_6_ units (and vice versa) via Mo2—Se1 bonds (and Mo1—Se2 bonds, respectively), forming the three-dimensional Mo—Se framework, the connectivity formula of which is Mo_9_Se*^i^*_5_Se*^i-a^*_6/2_Se*^-ai^*_6/2_, Mo_6_Se*^i^*_2_Se*^i-a^*_6/2_Se*^a-i^*_6/2_. It results from this arrangement that the shortest intercluster Mo1—Mo2 distance is 3.4216 (6) Å, indicating only weak metal—metal interaction. The In^+^ cations are surrounded by seven Se atoms forming a distorted tricapped tetrahedron, as is the case in In_2.9_Mo_15_Se_19_. The Se5 and Se2 atoms forming the tetrahedron are at 3.0759 (15) and 3.1221 (5) Å from the In atom, and the capping Se1 atoms are at 3.4904 (7) Å. The V^3+^ cations, as the In^3+^ cations in the In_3_Mo_15_Se_19_ compounds, occupy partially at 47.4 (6)% a triangular group of distorted octahedral cavities, which are formed by two Mo_6_Se*^i^*_8_Se*^a^*_6_ and three Mo_9_Se*^i^*_11_Se*^a^*_6_ units, around the threefold rotation axis. The V—Se distances are in the 2.510 (2) - 2.831 (3) Å range.

### Experimental

Single crystals of V_1.42_In_1.83_Mo_15_Se_19_ were prepared from a mixture of V_2_Se_3_, MoSe_2_, InSe and Mo with a nominal composition V_1.5_In_2_Mo_15_Se_19_. Before use, Mo powder was reduced under H_2_ flowing gas at 1273 K during ten hours in order to eliminate any trace of oxygen. The binaries V_2_Se_3_, MoSe_2_, InSe were obtained by heating stoichioetric mixtures of the elements in sealed evacuated silica tubes during about 2 days. All handlings of materials were done in an argon-filled glove box. The initial mixture (*ca*. 5 g) was cold pressed and loaded into a molybdenum crucible, which was sealed under a low argon pressure using an arc welding system. The charge was heated at the rate of 300 K/h up to 1773 K, the temperature which was held for 48 hours, then cooled at 100 K/h down to 1373 K and finally furnace cooled.

### Refinement

The highest peak and the deepest hole are located 1.56 Å and 0.66 Å from Mo3, respectively. Refinement of the occupancy factors of the V and In atoms led to the final composition V_1.42 (2)_In_1.832 (8)_Mo_15_Se_19_.

### Crystal data

**Table:**

V_1.42_In_1.83_Mo_15_Se_19_	*D*_x_ = 6.750 Mg m^−^^3^
*M**_r_* = 3221.80	Mo *K*α radiation, λ = 0.71069 Å
Hexagonal, *P*6_3_/*m*	Cell parameters from 26495 reflections
Hall symbol: -P 6c	θ = 2.6–35.0°
*a* = 9.7361 (1) Å	µ = 29.21 mm^−^^1^
*c* = 19.3090 (4) Å	*T* = 293 K
*V* = 1585.11 (4) Å^3^	Irregular block, black
*Z* = 2	0.09 × 0.07 × 0.05 mm
*F*(000) = 2797	

### Data collection

**Table:**

Nonius KappaCCD diffractometer	2390 independent reflections
Radiation source: fine-focus sealed tube	1634 reflections with *I* > 2σ(*I*)
graphite	*R*_int_ = 0.093
φ scans (κ = 0) + additional ω scans	θ_max_ = 35.0°, θ_min_ = 2.6°
Absorption correction: analytical (de Meulenaar & Tompa, 1965)	*h* = −15→15
*T*_min_ = 0.161, *T*_max_ = 0.329	*k* = −15→12
27560 measured reflections	*l* = −30→31

### Refinement

**Table:**

Refinement on *F*^2^	Primary atom site location: structure-invariant direct methods
Least-squares matrix: full	Secondary atom site location: difference Fourier map
*R*[*F*^2^ > 2σ(*F*^2^)] = 0.037	*w* = 1/[σ^2^(*F*_o_^2^) + (0.0413*P*)^2^ + 0.9587*P*] where *P* = (*F*_o_^2^ + 2*F*_c_^2^)/3
*wR*(*F*^2^) = 0.087	(Δ/σ)_max_ = 0.001
*S* = 1.08	Δρ_max_ = 3.22 e Å^−^^3^
2390 reflections	Δρ_min_ = −2.57 e Å^−^^3^
67 parameters	Extinction correction: *SHELXL97* (Sheldrick, 2008), Fc^*^=kFc[1+0.001xFc^2^λ^3^/sin(2θ)]^-1/4^
0 restraints	Extinction coefficient: 0.00025 (5)

### Special details

**Table:**

Geometry. All esds (except the esd in the dihedral angle between two l.s. planes) are estimated using the full covariance matrix. The cell esds are taken into account individually in the estimation of esds in distances, angles and torsion angles; correlations between esds in cell parameters are only used when they are defined by crystal symmetry. An approximate (isotropic) treatment of cell esds is used for estimating esds involving l.s. planes.
Refinement. Refinement of F^2^ against ALL reflections. The weighted R-factor wR and goodness of fit S are based on F^2^, conventional R-factors R are based on F, with F set to zero for negative F^2^. The threshold expression of F^2^ > 2sigma(F^2^) is used only for calculating R-factors(gt) etc. and is not relevant to the choice of reflections for refinement. R-factors based on F^2^ are statistically about twice as large as those based on F, and R- factors based on ALL data will be even larger.

### Fractional atomic coordinates and isotropic or equivalent isotropic displacement parameters (Å^2^)

**Table:**

	*x*	*y*	*z*	*U*_iso_*/*U*_eq_	Occ. (<1)
Mo1	0.16776 (5)	0.01669 (5)	0.55780 (2)	0.00914 (10)	
Mo2	0.68407 (5)	0.18577 (5)	0.63317 (2)	0.00965 (10)	
Mo3	0.51292 (7)	0.16694 (7)	0.7500	0.00868 (11)	
Se1	0.03647 (6)	−0.28702 (6)	0.55134 (3)	0.01070 (11)	
Se2	0.37798 (6)	0.00665 (6)	0.64065 (3)	0.01233 (12)	
Se3	0.34594 (8)	0.30753 (9)	0.7500	0.01280 (15)	
Se4	0.0000	0.0000	0.66221 (5)	0.01643 (19)	
Se5	0.6667	0.3333	0.52902 (5)	0.01225 (17)	
In1	0.6667	0.3333	0.36972 (6)	0.0374 (4)	0.916 (4)
V1	−0.2026 (4)	−0.1744 (3)	0.7500	0.0177 (9)	0.474 (6)

### Atomic displacement parameters (Å^2^)

**Table:**

	*U*^11^	*U*^22^	*U*^33^	*U*^12^	*U*^13^	*U*^23^
Mo1	0.00996 (18)	0.00853 (18)	0.0089 (2)	0.00456 (15)	0.00070 (14)	−0.00012 (14)
Mo2	0.00995 (18)	0.00993 (18)	0.0089 (2)	0.00488 (15)	0.00013 (14)	−0.00037 (14)
Mo3	0.0096 (2)	0.0099 (3)	0.0071 (3)	0.0053 (2)	0.000	0.000
Se1	0.0110 (2)	0.0097 (2)	0.0116 (2)	0.00543 (19)	0.00107 (17)	0.00229 (17)
Se2	0.0115 (2)	0.0119 (2)	0.0138 (3)	0.00598 (18)	−0.00305 (18)	−0.00208 (18)
Se3	0.0121 (3)	0.0158 (3)	0.0117 (3)	0.0079 (3)	0.000	0.000
Se4	0.0203 (3)	0.0203 (3)	0.0086 (4)	0.01016 (14)	0.000	0.000
Se5	0.0142 (2)	0.0142 (2)	0.0083 (4)	0.00712 (12)	0.000	0.000
In1	0.0345 (4)	0.0345 (4)	0.0431 (7)	0.0172 (2)	0.000	0.000
V1	0.0216 (16)	0.0095 (13)	0.0165 (16)	0.0038 (11)	0.000	0.000

### Geometric parameters (Å, °)

**Table:**

Mo2—Se5	2.5259 (8)	Mo3—Mo3^ii^	2.7056 (10)
Mo2—Se2	2.5974 (6)	Mo3—Mo3^i^	2.7056 (10)
Mo2—Se2^i^	2.6290 (6)	Mo3—Mo2^ix^	2.7136 (5)
Mo2—Mo2^i^	2.6474 (7)	Mo3—Mo2^ii^	2.7136 (5)
Mo2—Mo2^ii^	2.6474 (7)	Mo3—Mo2^viii^	2.7557 (5)
Mo2—Se1^iii^	2.6504 (6)	In1—Se5	3.0759 (15)
Mo2—Se3^i^	2.6966 (6)	In1—Se2^x^	3.1221 (5)
Mo2—Mo3^i^	2.7136 (5)	In1—Se2^xi^	3.1221 (5)
Mo2—Mo3	2.7557 (5)	In1—Se2^iv^	3.1221 (5)
Mo1—Se1^iv^	2.5467 (7)	In1—Se1^x^	3.4904 (7)
Mo1—Se4	2.5481 (8)	In1—Se1^xi^	3.4904 (7)
Mo1—Se1	2.5717 (6)	In1—Se1^iv^	3.4904 (7)
Mo1—Se1^v^	2.6111 (6)	In1—Se3^vii^	4.2444 (9)
Mo1—Se2	2.6378 (7)	In1—Se3^xii^	4.2444 (9)
Mo1—Mo1^vi^	2.6992 (7)	In1—Se3^xiii^	4.2444 (9)
Mo1—Mo1^v^	2.6992 (7)	V1—Se4	2.510 (2)
Mo1—Mo1^vii^	2.7223 (8)	V1—Se4^viii^	2.510 (2)
Mo1—Mo1^iv^	2.7223 (8)	V1—Se3^v^	2.730 (3)
Mo3—Se2	2.5631 (6)	V1—Se2^vi^	2.7981 (18)
Mo3—Se2^viii^	2.5631 (6)	V1—Se2^xiv^	2.7981 (18)
Mo3—Se3	2.5965 (9)	V1—Se3^vi^	2.831 (3)
Mo3—Se3^i^	2.6095 (9)	V1—Mo3^vi^	2.865 (3)
			
Se5—Mo2—Se2	92.603 (17)	Se2^viii^—Mo3—Mo3^i^	117.84 (2)
Se5—Mo2—Se2^i^	91.858 (17)	Se3—Mo3—Mo3^i^	118.92 (3)
Se2—Mo2—Se2^i^	173.59 (3)	Se3^i^—Mo3—Mo3^i^	58.45 (3)
Se5—Mo2—Mo2^i^	58.396 (13)	Mo3^ii^—Mo3—Mo3^i^	60.0
Se2—Mo2—Mo2^i^	120.06 (2)	Se2—Mo3—Mo2^ix^	149.88 (3)
Se2^i^—Mo2—Mo2^i^	58.978 (19)	Se2^viii^—Mo3—Mo2^ix^	59.685 (15)
Se5—Mo2—Mo2^ii^	58.396 (13)	Se3—Mo3—Mo2^ix^	60.991 (14)
Se2—Mo2—Mo2^ii^	60.16 (2)	Se3^i^—Mo3—Mo2^ix^	118.165 (16)
Se2^i^—Mo2—Mo2^ii^	118.878 (19)	Mo3^ii^—Mo3—Mo2^ix^	61.129 (16)
Mo2^i^—Mo2—Mo2^ii^	60.0	Mo3^i^—Mo3—Mo2^ix^	89.824 (14)
Se5—Mo2—Se1^iii^	90.42 (2)	Se2—Mo3—Mo2^ii^	59.685 (15)
Se2—Mo2—Se1^iii^	86.03 (2)	Se2^viii^—Mo3—Mo2^ii^	149.88 (3)
Se2^i^—Mo2—Se1^iii^	98.54 (2)	Se3—Mo3—Mo2^ii^	60.991 (14)
Mo2^i^—Mo2—Se1^iii^	137.757 (18)	Se3^i^—Mo3—Mo2^ii^	118.165 (16)
Mo2^ii^—Mo2—Se1^iii^	129.74 (2)	Mo3^ii^—Mo3—Mo2^ii^	61.129 (16)
Se5—Mo2—Se3^i^	175.68 (2)	Mo3^i^—Mo3—Mo2^ii^	89.824 (14)
Se2—Mo2—Se3^i^	85.76 (2)	Mo2^ix^—Mo3—Mo2^ii^	112.47 (3)
Se2^i^—Mo2—Se3^i^	89.46 (2)	Se2—Mo3—Mo2^viii^	145.46 (3)
Mo2^i^—Mo2—Se3^i^	119.150 (18)	Se2^viii^—Mo3—Mo2^viii^	58.328 (15)
Mo2^ii^—Mo2—Se3^i^	117.432 (18)	Se3—Mo3—Mo2^viii^	118.823 (15)
Se1^iii^—Mo2—Se3^i^	93.45 (2)	Se3^i^—Mo3—Mo2^viii^	60.271 (14)
Se5—Mo2—Mo3^i^	120.23 (2)	Mo3^ii^—Mo3—Mo2^viii^	88.941 (14)
Se2—Mo2—Mo3^i^	116.35 (2)	Mo3^i^—Mo3—Mo2^viii^	59.578 (16)
Se2^i^—Mo2—Mo3^i^	57.311 (17)	Mo2^ix^—Mo3—Mo2^viii^	57.893 (17)
Mo2^i^—Mo2—Mo3^i^	61.851 (15)	Mo2^ii^—Mo3—Mo2^viii^	146.06 (3)
Mo2^ii^—Mo2—Mo3^i^	91.063 (14)	Se2—Mo3—Mo2	58.328 (15)
Se1^iii^—Mo2—Mo3^i^	138.90 (2)	Se2^viii^—Mo3—Mo2	145.46 (3)
Se3^i^—Mo2—Mo3^i^	57.36 (2)	Se3—Mo3—Mo2	118.823 (15)
Se5—Mo2—Mo3	118.64 (2)	Se3^i^—Mo3—Mo2	60.271 (14)
Se2—Mo2—Mo3	57.123 (17)	Mo3^ii^—Mo3—Mo2	88.941 (14)
Se2^i^—Mo2—Mo3	116.58 (2)	Mo3^i^—Mo3—Mo2	59.578 (16)
Mo2^i^—Mo2—Mo3	90.142 (14)	Mo2^ix^—Mo3—Mo2	146.06 (3)
Mo2^ii^—Mo2—Mo3	60.255 (15)	Mo2^ii^—Mo3—Mo2	57.893 (17)
Se1^iii^—Mo2—Mo3	131.66 (2)	Mo2^viii^—Mo3—Mo2	109.90 (3)
Se3^i^—Mo2—Mo3	57.177 (19)	Mo1^vii^—Se1—Mo1	64.26 (2)
Mo3^i^—Mo2—Mo3	59.29 (2)	Mo1^vii^—Se1—Mo1^vi^	63.70 (2)
Se1^iv^—Mo1—Se4	176.34 (2)	Mo1—Se1—Mo1^vi^	62.77 (2)
Se1^iv^—Mo1—Se1	88.948 (17)	Mo1^vii^—Se1—Mo2^xv^	131.23 (2)
Se4—Mo1—Se1	91.765 (17)	Mo1—Se1—Mo2^xv^	128.19 (2)
Se1^iv^—Mo1—Se1^v^	88.084 (17)	Mo1^vi^—Se1—Mo2^xv^	81.114 (19)
Se4—Mo1—Se1^v^	90.859 (17)	Mo3—Se2—Mo2	64.549 (18)
Se1—Mo1—Se1^v^	173.79 (3)	Mo3—Se2—Mo2^ii^	63.004 (18)
Se1^iv^—Mo1—Se2	93.50 (2)	Mo2—Se2—Mo2^ii^	60.86 (2)
Se4—Mo1—Se2	90.13 (2)	Mo3—Se2—Mo1	130.39 (2)
Se1—Mo1—Se2	86.306 (19)	Mo2—Se2—Mo1	126.33 (2)
Se1^v^—Mo1—Se2	99.32 (2)	Mo2^ii^—Se2—Mo1	81.018 (19)
Se1^iv^—Mo1—Mo1^vi^	119.568 (17)	Mo3—Se3—Mo3^ii^	62.62 (3)
Se4—Mo1—Mo1^vi^	58.018 (12)	Mo3—Se3—Mo2^ix^	61.649 (16)
Se1—Mo1—Mo1^vi^	59.33 (2)	Mo3^ii^—Se3—Mo2^ix^	62.551 (16)
Se1^v^—Mo1—Mo1^vi^	117.827 (19)	Mo3—Se3—Mo2^ii^	61.649 (16)
Se2—Mo1—Mo1^vi^	129.11 (2)	Mo3^ii^—Se3—Mo2^ii^	62.551 (16)
Se1^iv^—Mo1—Mo1^v^	118.595 (17)	Mo2^ix^—Se3—Mo2^ii^	113.56 (3)
Se4—Mo1—Mo1^v^	58.018 (12)	Mo1^vi^—Se4—Mo1	63.96 (2)
Se1—Mo1—Mo1^v^	119.25 (2)	Mo1^vi^—Se4—Mo1^v^	63.96 (2)
Se1^v^—Mo1—Mo1^v^	57.903 (19)	Mo1—Se4—Mo1^v^	63.96 (2)
Se2—Mo1—Mo1^v^	137.270 (18)	Mo2^i^—Se5—Mo2	63.21 (3)
Mo1^vi^—Mo1—Mo1^v^	60.0	Mo2^i^—Se5—Mo2^ii^	63.21 (3)
Se1^iv^—Mo1—Mo1^vii^	59.30 (2)	Mo2—Se5—Mo2^ii^	63.21 (3)
Se4—Mo1—Mo1^vii^	118.273 (15)	Mo2^i^—Se5—In1	142.763 (16)
Se1—Mo1—Mo1^vii^	57.423 (15)	Mo2—Se5—In1	142.763 (16)
Se1^v^—Mo1—Mo1^vii^	116.42 (2)	Mo2^ii^—Se5—In1	142.763 (16)
Se2—Mo1—Mo1^vii^	132.182 (19)	Se4^viii^—V1—Se4	84.98 (9)
Mo1^vi^—Mo1—Mo1^vii^	60.281 (10)	Se4^viii^—V1—Se3^v^	87.27 (7)
Mo1^v^—Mo1—Mo1^vii^	90.0	Se4—V1—Se3^v^	87.27 (7)
Se1^iv^—Mo1—Mo1^iv^	58.31 (2)	Se4^viii^—V1—Se2^vi^	166.76 (13)
Se4—Mo1—Mo1^iv^	118.273 (15)	Se4—V1—Se2^vi^	87.36 (2)
Se1—Mo1—Mo1^iv^	116.86 (2)	Se3^v^—V1—Se2^vi^	103.16 (8)
Se1^v^—Mo1—Mo1^iv^	56.998 (14)	Se4^viii^—V1—Se2^xiv^	87.36 (2)
Se2—Mo1—Mo1^iv^	140.74 (2)	Se4—V1—Se2^xiv^	166.76 (13)
Mo1^vi^—Mo1—Mo1^iv^	90.0	Se3^v^—V1—Se2^xiv^	103.16 (8)
Mo1^v^—Mo1—Mo1^iv^	60.281 (10)	Se2^vi^—V1—Se2^xiv^	97.98 (8)
Mo1^vii^—Mo1—Mo1^iv^	59.44 (2)	Se4^viii^—V1—Se3^vi^	85.09 (8)
Se2—Mo3—Se2^viii^	110.93 (3)	Se4—V1—Se3^vi^	85.09 (8)
Se2—Mo3—Se3	93.18 (2)	Se3^v^—V1—Se3^vi^	169.63 (12)
Se2^viii^—Mo3—Se3	93.18 (2)	Se2^vi^—V1—Se3^vi^	83.50 (6)
Se2—Mo3—Se3^i^	88.30 (2)	Se2^xiv^—V1—Se3^vi^	83.50 (6)
Se2^viii^—Mo3—Se3^i^	88.30 (2)	Se4^viii^—V1—Mo3^vi^	122.97 (8)
Se3—Mo3—Se3^i^	177.38 (3)	Se4—V1—Mo3^vi^	122.97 (8)
Se2—Mo3—Mo3^ii^	120.789 (19)	Se3^v^—V1—Mo3^vi^	136.14 (12)
Se2^viii^—Mo3—Mo3^ii^	120.789 (19)	Se2^vi^—V1—Mo3^vi^	53.80 (4)
Se3—Mo3—Mo3^ii^	58.92 (3)	Se2^xiv^—V1—Mo3^vi^	53.80 (4)
Se3^i^—Mo3—Mo3^ii^	118.45 (3)	Se3^vi^—V1—Mo3^vi^	54.23 (5)
Se2—Mo3—Mo3^i^	117.84 (2)		

Symmetry codes: (i) −*y*+1, *x*−*y*, *z*; (ii) −*x*+*y*+1, −*x*+1, *z*; (iii) −*x*+*y*+1, −*x*, *z*; (iv) *x*−*y*, *x*, −*z*+1; (v) −*y*, *x*−*y*, *z*; (vi) −*x*+*y*, −*x*, *z*; (vii) *y*, −*x*+*y*, −*z*+1; (viii) *x*, *y*, −*z*+3/2; (ix) −*x*+*y*+1, −*x*+1, −*z*+3/2; (x) −*x*+1, −*y*, −*z*+1; (xi) *y*+1, −*x*+*y*+1, −*z*+1; (xii) *x*−*y*+1, *x*, −*z*+1; (xiii) −*x*+1, −*y*+1, −*z*+1; (xiv) −*x*+*y*, −*x*, −*z*+3/2; (xv) −*y*, *x*−*y*−1, *z*.
